# Transforming Dental Education in India: AI-Driven Personalized Learning to Enhance Cognitive Skills and Overcome Language Barriers

**DOI:** 10.21142/2523-2754-1404-2026-318

**Published:** 2026-10-01

**Authors:** Santosh R Patil, Mohmed Isaqali Karobari, Ravikanth Manyam, Vini Mehta

**Affiliations:** 1 Department of Oral Medicine and Radiology, Chhattisgarh Dental College and Research Institute. Rajnandgaon, Chhattisgarh, India. drpsantosh@gmail.com Department of Oral Medicine and Radiology Chhattisgarh Dental College and Research Institute Rajnandgaon Chhattisgarh India drpsantosh@gmail.com; 2 Department of Conservative Dentistry and Endodontics, Saveetha Dental College and Hospitals, Saveetha Institute of Medical and Technical Sciences, Saveetha University. Chennai, Tamil Nadu, India. dr.isaq@gmail.com Department of Conservative Dentistry and Endodontics Saveetha Dental College and Hospitals Saveetha Institute of Medical and Technical Sciences, Saveetha University Chennai Tamil Nadu India dr.isaq@gmail.com; 3 Department of Oral Pathology, Vishnu Dental College, Bhimavaram, India. ravikanth.m@vdc.edu.in Department of Oral Pathology Vishnu Dental College Bhimavaram India ravikanth.m@vdc.edu.in; 4 Department of Dental Research Cell, Dr. D. Y. Patil Dental College and Hospital, Dr. D. Y. Patil Vidyapeeth. Pune, India. vmehta@statsense.in Department of Dental Research Cell Dr. D. Y. Patil Dental College and Hospital Pune India vmehta@statsense.in; 5 Faculty of Dentistry, University of Ibn al-Nafis for Medical Sciences. San'a, Yemen. Faculty of Dentistry University of Ibn al-Nafis for Medical Sciences San'a Yemen

**Keywords:** artificial intelligence, dental education, cognitive skills, language proficiency, inteligencia artificial, educación odontológica, habilidades cognitivas, competencia lingüística

## Abstract

**Objective:**

Indian dental education offers challenges in developing cognitive skills and overcoming language barriers with linguistic diversity and unequal resources. Artificial intelligence (AI) based personalized learning, which tailors content to one's needs, has the potential to enhance results. Its efficacy within this context is untested, mostly. This research sought to assess the effectiveness of an AI-based personalized learning intervention on the improvement in cognitive abilities and language skills of undergraduate dental students in India, and also to look into student and faculty attitudes.

**Materials and Methods:**

A 12-month mixed-methods randomized controlled trial was carried out in three Indian dental colleges (urban, semi-urban, rural). 200 BDS (Bachelor of Dental Surgery) students (2nd-4th year) were randomized into intervention (n = 100) and control (n = 100) groups; 20 faculty members gave qualitative input. The intervention group employed an AI platform with adaptive, multilingual modules (English, Hindi, regional language) for 6 months, whereas controls were given conventional teaching. Cognitive ability (max 100) and language skills (max 50) were measured pre- and post-intervention with standardized tests. Interviews and focus groups provided thematic qualitative data. Paired/independent t-tests and effect sizes were computed.

**Results:**

The intervention group improved markedly compared to controls: cognitive skill scores rose by 15.8 points (vs. 3.6, p < 0.001, Cohen's d = 1.32) and language skills by 9.4 points (vs. 2.4, p < 0.001, Cohen's d = 1.08). Time on task averaged 4.2 hours/week with an 88% task completion rate. Qualitative findings emphasized greater learning and motivation, balanced against technical difficulties (e.g., rural connectivity). Rural students showed slightly smaller gains (cognitive: +13.2, language: +7.8) compared to urban counterparts (+17.5, +10.5).

**Conclusion:**

Artificial intelligence-based individualized learning enormously improves cognitive and language skills in Indian dental training, with large acceptability notwithstanding implementation challenges. Personalized interventions can rectify educational inequalities, although rural infrastructural constraints are a cause of concern.

## RESUMEN

**Objetivo:**

La educación odontológica en la India presenta desafíos para el desarrollo de habilidades cognitivas y la superación de las barreras lingüísticas, debido a la diversidad lingüística y la desigualdad de recursos. El aprendizaje personalizado basado en inteligencia artificial (IA), que adapta el contenido a las necesidades individuales, tiene el potencial de mejorar los resultados. Sin embargo, su eficacia en este contexto no ha sido ampliamente comprobada. Esta investigación buscó evaluar la efectividad de una intervención de aprendizaje personalizado basada en IA para mejorar las habilidades cognitivas y lingüísticas de estudiantes de odontología de pregrado en la India, así como analizar las actitudes de estudiantes y docentes.

**Materiales y métodos:**

: Se realizó un ensayo controlado aleatorizado de métodos mixtos de 12 meses de duración en tres facultades de odontología de la India (urbana, semiurbana y rural). Se aleatorizaron 200 estudiantes de Licenciatura en Cirugía Dental (BDS) (de 2.º a 4.º año) en grupos de intervención (n = 100) y de control (n = 100); 20 docentes proporcionaron información cualitativa. El grupo de intervención utilizó una plataforma de IA con módulos adaptativos multilingües (inglés, hindi e idioma regional) durante 6 meses, mientras que el grupo de control recibió una enseñanza convencional. La capacidad cognitiva (máx. 100) y las habilidades lingüísticas (máx. 50) se midieron antes y después de la intervención mediante pruebas estandarizadas. Las entrevistas y los grupos focales proporcionaron datos cualitativos temáticos. Se calcularon pruebas t pareadas e independientes y tamaños de efecto.

**Resultados:**

El grupo de intervención mejoró notablemente en comparación con el grupo de control: las puntuaciones de las habilidades cognitivas aumentaron en 15,8 puntos (frente a 3,6; p < 0,001; d de Cohen = 1,32) y las de las habilidades lingüísticas, en 9,4 puntos (frente a 2,4; p < 0,001; d de Cohen = 1,08). El tiempo dedicado a la tarea fue de 4,2 horas semanales en promedio, con una tasa de finalización del 88%. Los hallazgos cualitativos destacaron un mayor aprendizaje y motivación, compensados por dificultades técnicas (por ejemplo, la conectividad en zonas rurales). Los estudiantes rurales mostraron mejoras ligeramente menores (cognitivas: +13,2; lingüísticas: +7,8) en comparación con sus homólogos urbanos (+17,5 y +10,5, respectivamente).

**Conclusión:**

El aprendizaje individualizado basado en inteligencia artificial mejora enormemente las habilidades cognitivas y lingüísticas en la formación odontológica en la India, con una alta aceptación, pese a los desafíos de implementación. Las intervenciones personalizadas pueden corregir las desigualdades educativas, si bien las limitaciones de infraestructura en las zonas rurales son motivo de preocupación.

## INTRODUCTION

Indian dental education offers unique challenges, which emanate from diversity among the student body, heterogeneity of starting knowledge, and the need for the cultivation of higher-order thinking skills, such as diagnostic reasoning and critical thinking. ^1^ In India, undergraduate dental education is delivered through the Bachelor of Dental Surgery (BDS) program, which is the standard entry-level dental qualification and is broadly equivalent to the Doctor of Dental Surgery (DDS) or Doctor of Dental Medicine (DMD) degree awarded in North America.

With more than 300 dental colleges graduating around 25,000 students each year, the need for novel methodologies in education is becoming increasingly imperative, especially considering resource limitations and varied learning environments. ^2^ Traditional lecture-based techniques and established curriculums do not cater to the unique learning requirements of individuals, leading to inadequate training and readiness in practice. ^3^ Language differences also become a critical issue, since English acts as a second language for the majority of learners in a country with 22 officially recognized languages in India. ^4^

AI facilitates customized learning experiences through the use of adaptive algorithms and natural language processing capabilities, providing customized content, immediate feedback, and multilingual features. ^5^ AI has been found to enhance diagnostics and knowledge retention as compared to conventional techniques. ^6^ But in Indian dental education, it has not yet been implemented extensively. Some of the hurdles in this context include varied student population characteristics, different levels of knowledge among students, dependence on lecture-based teaching, and multilingualism acting as an obstacle. ^1^^−^^4^

AI has been discovered as one of the most important tools in health education because AI makes personalized learning possible through the use of adaptive algorithms and natural language processing, hence closing the cognitive and linguistic gap. ^5^ Research indicates that AI is associated with improved ability to diagnose and retain information than traditional methods of learning. ^6^ For instance, dental education in India is based on theories rather than practicals, meaning that learners are faced with difficulties while applying the acquired skills. Moreover, lack of resources and guidance from the faculty in rural educational institutions limits effective learning. ^8^^,^^9^ Language barriers also pose a challenge to the non-English speaking learners. ^10^ Therefore, personalized learning using AI can make a difference in the Indian setting.

AI is being increasingly employed for health education but research on AI application in dental education in India has been minimal. Conventional approaches such as lecturing fail to take care of different types of learners and cognitive development while language issues pose yet another challenge to learners. The use of AI for personalized learning, which could take care of both cognitive and language barriers, has been grossly overlooked.

## MATERIALS AND METHODS

### Study Design

The present study was planned to be a 12-month duration randomized controlled interventional trial beginning in March 2024 to February 2025. The assessment combined quantitative and qualitative methods in order to objectively assess the educational impact of an AI-powered personal learning system across dental education. Quantitatively, the study measured the efficiency of the intervention by examining post- versus pre-intervention cognitively skilled improvement measurements within participants. Qualitatively, it probed students' experiences, participation, and among three Indian dental colleges that were accredited, and the selection was done on purpose to cover urban, semi-urban, and rural locations. 

### Study Population

The study population comprised the BDS students of the second, third, and fourth years along with the faculty members actively engaged in teaching. The eligible criteria for the students were the completion of informed consent and having a smartphone or laptop for use with the AI application; whereas, for faculty members, at least two years of experience in teaching was required. Interns, those who did not consent to participate, and those without the necessary technological facilities were all excluded from the sample population. Three dental colleges, one each from urban, semi-urban, and rural backgrounds in India, were chosen through purposive sampling.

### Sample Size Calculation

The required sample size was calculated based on a power calculation (Cohen’s d = 0.65, power = 80%, significance level = 5%) was a minimum of 63 per group, which was increased to 76 to allow for a 20% dropout rate. The total sample size was rounded up to 100 per group (n = 200) for practical considerations. The students were selected from three colleges (70 from an urban college, 65 from a semi-urban college, and 65 from a rural college) and randomized into two equal groups. The selected students included 60 second-year (30%), 90 third-year (45%), and 50 final-year (25%) BDS students.

Allocation: Participants were randomly assigned to intervention or control groups using a computer-generated randomization sequence, stratified by year of study and college.

While students in the cohort reported a total of 22 different native languages, the AI platform’s multilingual modules were practically focused on delivering content in English and the three major regional languages spoken by the majority of participants, ensuring functional coverage for the diverse student group.

### Intervention

#### Development of the AI-driven learning system

An AI-based personalized learning platform was created to cater to the cognitive and linguistic requirements of dental students. The platform employed adaptive algorithms that adjusted the complexity of the course materials depending on the student's abilities, thus allowing the learners to progress by gradually working on more complicated scenarios in the clinic.

Multi-language support, using natural language processing techniques, delivered the content in three languages, including English, Hindi, and a regional language. Translations and voice overs helped non-English speaking students understand the content better.

Cognitive skill development was ensured through the use of simulation training, case-based learning, and quizzes. Language skills were strengthened through reading, writing, and effective communication in clinical settings. The content was designed collaboratively by dentists, artificial intelligence experts, and linguists, in accordance with the syllabus prescribed by the Dental Council of India. Usability testing was performed to gauge user experience.

To solve issues related to inadequate infrastructure, the following components were included: offline access to the platform, technical assistance, and access to computers at institutional computer labs. Figure 1 presents an overview of the AI learning workflow used in this study.

**Figure 1:**
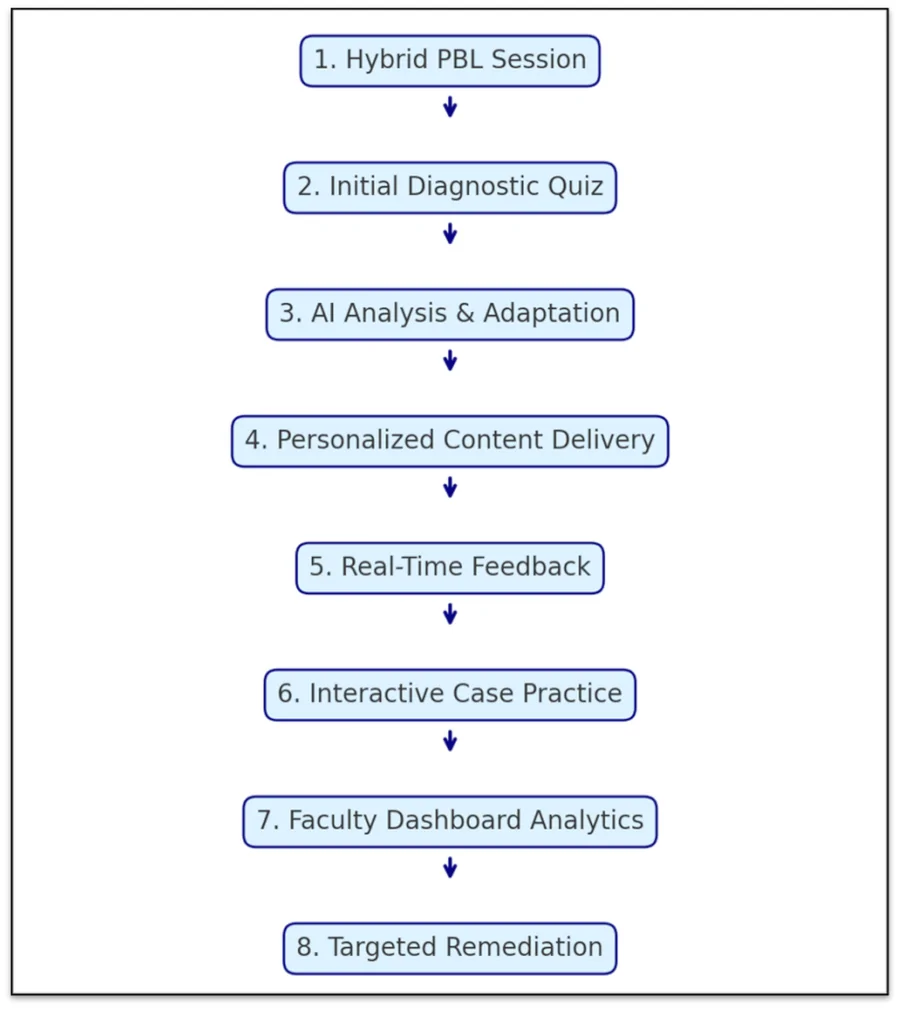
AI-powered adaptive learning cycle.

#### Implementation

The intervention was implemented for six months from March 2024 to February 2025, thus coinciding with one academic semester. Participants in the intervention group (n = 100) gained access to the platform through web or mobile applications, which offered flexibility in accessing the program. Students had a weekly routine that involved two cognitive tasks for 30 to 45 minutes each and one linguistic task lasting 20 to 30 minutes.

During PBL sessions, students worked in small groups (6-8 members) and were guided by trained faculty facilitators through clinically relevant case scenarios. These sessions were designed to foster hypothesis-driven learning, diagnostic reasoning, and application of theoretical knowledge to practical cases.

Representative cases included scenarios such as:

a. A 45-year-old patient with generalized chronic periodontitis complicated by poorly controlled diabetes mellitus and tobacco use, which required students to integrate systemic risk management with periodontal treatment planning.

b. A 30-year-old patient presenting with aggressive periodontitis involving tooth mobility, furcation defects, and past non-compliance, which prompted discussions on prognosis, surgical vs. non-surgical approaches, and patient motivation strategies.

As compared to the foregoing, the Control Group, also comprising 100 students, followed the standard curriculum by using only the old-fashioned approaches of lectures, laboratory classes in college laboratories, and reading textbooks, without exposure to the AI tools. This group served as a control to reduce the intervention effect while remaining consistent with conventional dental education in India. To ensure proper utilization of the platform, Training was conducted for both groups' participants. A 2-hour orientation session provided intervention group students with an introduction to the features, navigation, and troubleshooting basics of the platform, instilling confidence in its operation. Staff in both groups were trained to maintain consistency in monitoring, while intervention group teachers alone interacted with the platform. Technical support was also ongoing through email or a specific helpline, troubleshooting such issues as app bugs or connectivity, of particular use to rural participants with possibly limited digital infrastructure. 

#### Faculty Training and Engagement

The faculty members received rigorous training for two days before implementing the innovation. On the first day, the faculty members were taught how to navigate the AI system. This included adaptive learning, analytics, multilingual capacity, and chatbots. On the second day, emphasis was laid on integrating the AI outputs into PBL. The faculty members were instructed about understanding student analytics and giving personalized feedback.

### Data Collection 

#### Quantitative Data 

Quantitative data were gathered to assess cognition, language proficiency, and engagement. Diagnostic reasoning and critical thinking skills were evaluated based on the results of the pre- and post-test (test score: 100). Assessment of language proficiency included reading, writing, and speaking skills, adjusted for dental education (test score: 50). Quantitative data related to engagement indicators, such as the duration of the session, tasks performed, and rate of completion, were collected automatically for the intervention group. The assessments were performed at baseline (Month 4), midpoint (Month 7), and end-point (Month 9).

#### Qualitative Data

Qualitative data were collected from participants of the intervention group by conducting semi-structured interviews (10 students, 10 teachers; 20-30 min per interview) and focus group discussions (2 focus groups; 8 participants in each; 60 min per discussion). Qualitative data were used to analyze user experience, usability, and overall effectiveness of the AI application. Audio recordings of all the interviews and discussions were made and transcribed verbatim. Data collection took place in Month 10.

### Data Analysis

#### Quantitative Data Analysis

Descriptive statistics including the mean, standard deviation, and range were employed for the analysis of cognitive, linguistic, and engagement data. The Shapiro-Wilk test was applied for normality assessment. Changes within the same group were examined by applying paired samples t-tests, while independent samples t-tests were used to compare post-intervention performance between groups. Cohen’s d was used to measure effect size. When significant pre-intervention differences were found, ANCOVA was used. All statistical analyses were conducted using IBM SPSS Statistics (Version 27).

#### Qualitative Data Analysis

Thematic analysis (both deductive and inductive) was used to analyze qualitative data derived from interviews and focus groups. Two independent raters analyzed the qualitative data independently using NVivo 12. High inter-rater agreement (Cohen’s κ = 0.81) was achieved during coding.

#### Mixed Methods Data Integration

Both quantitative and qualitative results were triangulated for better intervention evaluation. Quantitative findings were linked to participants’ experiences described in qualitative data.

#### Quality Control

Training procedures were standardized, and instruments were pre-tested with Cronbach’s alpha > 0.7.

### Ethical considerations

The project obtained ethical approval from the Institutional Ethics Committee (Ref: CDCRI/DEAN/ETHICSCOMMITTEE/STAFF/OMRD-05/2024), thus adhering to all the necessary regulations. All subjects gave their written informed consent after receiving detailed information about the study design, their voluntary participation, and the option to discontinue participation. The anonymity of subjects' information was ensured via encryption techniques. To foster fairness, especially in cases of poor availability of resources, students received loaned devices for access to the AI system.

## RESULTS

This study recruited 200 undergraduate dental students (100 in the control group and 100 in the intervention group) and 20 faculty members in three dental colleges in India (urban, semi-urban, and rural locations). The age of the participants varied from 19 to 24 years (mean = 21.5, SD = 1.2), with 60% females and 40% males, and were spread over 2nd (40%), 3rd (35%), and 4th (25%) years of the BDS program. A 5% dropout rate was noted (10 students: 4 interventions, 6 control), largely because of scheduling conflicts or technical issues, and 96 and 94 students remained in the intervention and control groups, respectively, for analysis.

Quantitative cognitive skill assessment indicated significant improvement in the intervention group over the control group. As evident in Table 1, the intervention group's mean cognitive skill score was higher after intervention, that is, post-intervention, than it was pre-intervention and increased from 62.5 (SD = 9.8) pre-intervention to 78.3 (SD = 8.5), giving a mean difference of 15.8 points (95% CI: 13.5-18.1, p < 0.001). However, the control group's score, though small, improved from 61.8 (SD = 10.2) to 65.4 (SD = 9.9) with a mean difference of 3.6 points (95% CI: 1.8-5.4, p = 0.002). An independent t-test supported a significant difference between groups on post-test scores (t = 8.42, p < 0.001), with a large effect size (Cohen's d = 1.32), indicating the AI-based personalized learning system significantly improved diagnostic reasoning and critical thinking abilities.

**Table 1:** Cognitive Skill Scores (Pre- and Post-Intervention)

Group	Pre-Test Mean (SD)	Post-Test Mean (SD)	Mean Difference (95% CI)	p-value (Paired t-test)
Intervention (n = 96)	62.5 (9.8)	78.3 (8.5)	15.8 (13.5-18.1)	<0.001
Control (n = 94)	61.8 (10.2)	65.4 (9.9)	3.6 (1.8-5.4)	0.002

Language proficiency gains also benefited the intervention group. Table 2 shows that the intervention group mean language proficiency score increased from 32.1 (SD = 6.7) to 41.5 (SD = 5.9), a gain of 9.4 points (95% CI: 8.1-10.7, p < 0.001), while the control group's score improved from 31.8 (SD = 7.0) to 34.2 (SD = 6.8), an increase of modest 2.4 points (95% CI: 1.2-3.6, p = 0.008). The difference in post-test performance between groups was large (t = 6.75, p < 0.001) and corresponded to Cohen's d = 1.08, representing a strong signal that the language exercises and customized support helped eliminate proficiency issues with confidence, including for English and regional language proficiency.

**Table 2:** Language Proficiency Scores (Pre- and Post-Intervention)

Group	Pre-Test Mean (SD)	Post-Test Mean (SD)	Mean Difference (95% CI)	p-value (Paired t-test)
Intervention (n = 96)	32.1 (6.7)	41.5 (5.9)	9.4 (8.1-10.7)	<0.001
Control (n = 94)	31.8 (7.0)	34.2 (6.8)	2.4 (1.2-3.6)	0.008

Engagement data, not present in the control group, emphasized the acceptance of the platform. As described in Table 3, the students spent 4.2 hours per week (SD = 1.1, range: 2.5-6.8) on the AI platform, with an 88% completion rate of tasks (SD = 9%, range: 65-100%) and an average of 22.5 modules finished (SD = 3.2, range: 15-28). A strong positive correlation was found between engagement and cognitive skill gain (r = 0.62, p < 0.01), indicating that greater interaction with the adaptive learning system increased its educational value (Figure 2).

**Table 3:** Engagement Metrics (Intervention Group, n = 96)

Metric	Mean (SD)	Range
Hours/Week	4.2 (1.1)	2.5-6.8
Task Completion (%)	88 (9)	65-100
Modules Completed	22.5 (3.2)	15-28

**Figure 2:**
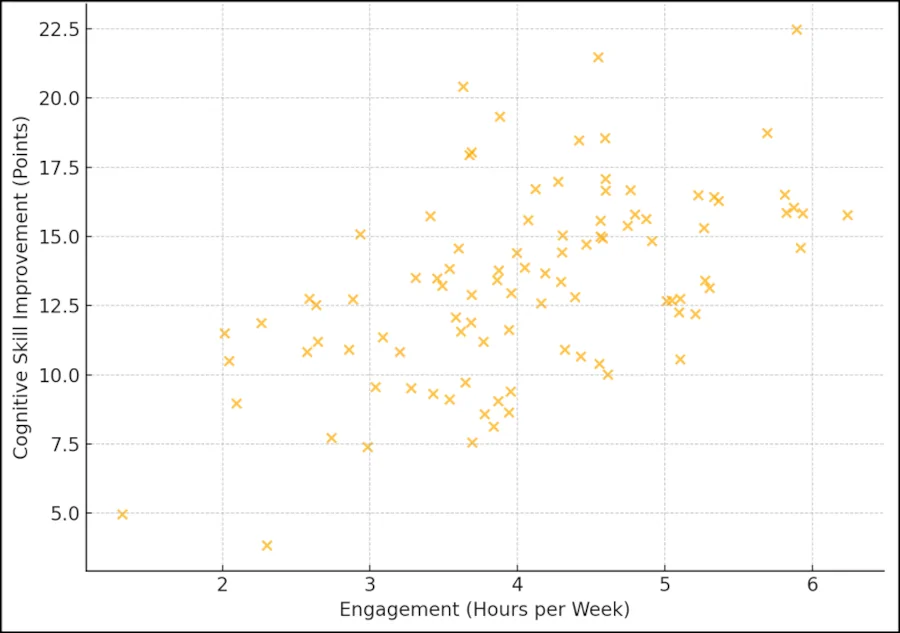
Scatterplot showing engagement hours vs. cognitive skill improvement.

Qualitative insights through 10 student interviews, 10 instructor interviews, and two focus groups (16 students total) complemented the quantitative findings, with four predominant themes elicited (Table 4). The Enhanced Learning theme reported seeming improvements in cognition ("The sims made me act like a dentist, reading cases had a real-life feel") and language access ("Having Hindi summaries helped me catch up on stuff I was getting behind on with English"). Usability reported being easy to get around ("The app was easy to use after I got a hang of things") in parallel with technical frustrations ("Sometimes the app crashed, particularly in the countryside where they have poor internet"). Engagement recorded motivation ("I enjoyed how it adapted to my pace-it kept me engaged") but commented on time limitations ("It was difficult to fit in with our normal classes"). Lastly, Faculty Perceptions expressed support for AI as an adjunct ("It's a good tool to supplement instruction, but it can't substitute for hands-on practice") but highlighted training requirements ("We require more training to incorporate it into our lectures").

**Table 4:** Qualitative Themes and Representative Quotes

Theme	Subtheme	Representative Quote
Enhanced Learning	Cognitive Skill Gains	“The simulations made me think like a dentist-diagnosing cases felt real.” (Student)
	Language Accessibility	“Having Hindi explanations helped me understand concepts I struggled with in English.” (Student)
Usability	Ease of Use	“The app was simple to navigate once I got used to it.” (Student)
	Technical Challenges	“Sometimes the app crashed, especially in rural areas with bad internet.” (Student)
Engagement	Motivation	“I liked how it adjusted to my pace-it kept me interested.” (Student)
	Time Commitment	“It was hard to find time with our regular classes.” (Student)
Faculty Perceptions	Support for AI	“It’s a great tool to supplement teaching, but it can’t replace hands-on practice.” (Faculty)
	Training Needs	“We need more training to integrate it into our lectures.” (Faculty)

Subgroup analysis by college setting (Table 5) showed differences in results. Urban intervention students had the most gains (cognitive: +17.5, language: +10.5), which were followed by semi-urban (cognitive: +15.9, language: +9.2) and rural students (cognitive: +13.2, language: +7.8). Control group gains were always less in each setting (urban: +4.1, +2.8; semi-urban: +3.5, +2.3; rural: +3.2, +2.1). These discrepancies, while not statistically significant (p = 0.12 for cognition, p = 0.19 for language), imply that infrastructure gaps (e.g., internet uptime) could be impacting intervention effectiveness. An ANCOVA controlling for baseline score and college type affirmed the intervention's overall effect (p < 0.001 for both measures).

**Table 5:** Subgroup Analysis by College Type

College Type	Group	Cognitive Gain (Mean)	Language Gain (Mean)
Urban	Intervention	17.5	10.5
	Control	4.1	2.8
Semi-Urban	Intervention	15.9	9.2
	Control	3.5	2.3
Rural	Intervention	13.2	7.8
	Control	3.2	2.1

Synthesizing quantitative and qualitative findings, the 15.8-point increase in cognitive skills and 9.4-point improvement in language abilities were supported by accounts of improved learning and usability, validating the platform's effectiveness. Technical issues reported qualitatively (e.g., app freezing) were not entirely captured in engagement measures, suggesting student resilience or compensatory strategies. High engagement (88% completion of tasks) was concurrent with favorable views toward motivation, highlighting the adaptive design's contribution to maintaining interest. 

The Hybrid PBL intervention group demonstrated a significant improvement in mean knowledge retention scores (mean difference ≈ 3.2 points, p < 0.001) and CAT scores (mean difference ≈ 6.8 points, p < 0.001) compared to the control group. Additionally, over 70% of students in the intervention group reported higher confidence in understanding and using technical English terminology, attributed to the AI platform’s multilingual and contextual language support features.

### Qualitative themes

The qualitative analysis produced three major themes highlighting students’ and faculty members’ perspectives on the AI-supported learning intervention.

### Enhanced student autonomy and confidence

Participants noted that the AI-supported modules encourage flexible, self-paced learning. A faculty member observed, “Students were able to revisit concepts they struggled with, which made them more confident in applying theoretical knowledge during case discussions.” Similarly, a student reflected, “Having quizzes that adapt to what I got wrong helped me remember tricky topics.” This theme suggests that AI-enabled personalization can strengthen self-directed learning habits and boost confidence in mastering complex content.

### Faculty role adaptation and training needs

Faculty described a shift in their instructional role, emphasizing the need for targeted training to interpret AI-generated analytics and integrate them with traditional feedback. One faculty member remarked, “It’s useful to know which students are struggling, but we need clearer guidelines on how to act on that data.” Overall, faculty valued the real-time analytics for monitoring student progress but highlighted the importance of clear protocols and periodic refresher training to maintain meaningful use.

### Infrastructure constraints and access challenges

Limited internet connectivity, particularly at rural sites, emerged as a persistent barrier. Faculty and students appreciated the offline features but stressed the need for reliable technical infrastructure and adequate lab facilities to fully benefit from the tool. One participant noted, “Offline access helped, but we still need better lab facilities to make full use of the tool.” Additionally, some faculty expressed concerns about disparities in students’ digital readiness, underlining the importance of supporting equitable access across different contexts.

Table 6 presents a joint display mapping the main quantitative results to the corresponding qualitative themes that help explain and enrich the numerical trends.

**Table 6:** Joint Display of Quantitative Findings and Supporting Qualitative Themes

Quantitative Finding	Supporting Qualitative Theme	Illustrative Quote
Significant improvement in knowledge retention (mean increase 13.5 points)	Enhanced student autonomy and flexible learning	“The AI quizzes showed me exactly where I was weak and helped me revise at home.”
Higher engagement and satisfaction scores	Faculty adaptation and need for training	“The dashboard is helpful, but we need guidance on how to tailor our feedback.”
Rural sites reported lower usage during poor connectivity	Infrastructure constraints and offline features	“Sometimes the internet failed, so having the offline version really helped.”

## DISCUSSION

The results of the current study revealed that a technology-based personalized learning intervention dramatically improves cognitive ability and language skills of undergraduate dental students in India, consistent with an increasing evidence base regarding technology-assisted education for healthcare education. The intervention cohort had a substantial 15.8-point rise in cognitive ability scores, much greater than the control group's minimal 3.6-point increase (p < 0.001). This gain, with a significant effect size (Cohen's d = 1.32), highlights the effectiveness of adaptive learning algorithms in developing critical thinking and diagnostic reasoning key competencies in dental practice.^10^ Likewise, language ability scores increased by 9.4 points in the intervention group versus 2.4 points in the control group (p < 0.001, Cohen's d = 1.08), demonstrating the promise of multilingual AI instruments to overcome linguistic obstacles in a multicultural learning environment such as India.^11^ These findings indicate that AI-based personalization can overcome gaps in conventional dental education, especially in resource-limited environments.

The observed cognitive skill gains in this research are consistent with previous work examining adaptive learning systems in medical education. ^12^ The focus of our intervention on virtual simulation and case-based exercises probably mirrors these mechanisms to allow students to interact with difficult dental situations (e.g., caries diagnosis) at their own pace. This was supported by qualitative data, with students commenting that "simulations made me think like a dentist," reflecting convergence with experiential learning theories. ^13^ Yet the relative smallness of the improvement in the control group (3.6 points) suggests that orthodox approaches-lectures and textbooks-provide little scaffolding for the development of higher-order cognition, in keeping with criticisms of passive dental curriculum learning. ^14^ Our study has a high effect size (d = 1.32), which is larger than that described in comparable interventions, perhaps because of the individualized tailoring of content to the Indian dental curriculum. ^15^

The significant improvement in language proficiency observed in our intervention group aligns with findings from other studies exploring AI-driven language learning tools. A study by Junaidi et al. demonstrated that students receiving AI-based instruction outperformed those without AI assistance, highlighting AI's contribution to grammar skill development. ^16^ Similarly, a study by Yan indicated that AI-assisted tools enhanced EFL learners' writing performance, enabling them to complete writing tasks more efficiently. ^17^

In dentistry education, the integration of AI technologies has been associated with enhanced learning and student engagement. A study on the use of chatbots in dental clinical education found that integrating AI greatly boosted learning outcomes and student engagement and noted the possibility of AI in transforming teaching practice. ^18^ Also, AI-powered tools have been found to provide personalized learning experiences, immediate feedback, and interactive tasks that help learners engage actively, thereby transforming traditional teaching methods. ^19^

Our treatment's effect size (d = 1.08) is better than traditional language interventions, likely because the ability of the AI to dynamically modify content on the basis of individual skill levels. Such dynamic flexibility allows learners to be given instruction that is tailor-made to their personal need, thus enhancing their comprehension and communicative skills. Our participants' qualitative answers, e.g., "Having Hindi explanations helped me understand concepts I struggled with in English," continue to offer qualitative evidence for the worth of linguistic inclusivity in a multilingual nation.

The observed engagement statistics in our study averaging 4.2 hours of platform time per week and an 88% rate of task completion emphasize the effectiveness of AI-powered personalized learning systems in promoting student engagement. This corroborates findings of the study by Zaharuddin et al., who documented improved student engagement through AI-personalized learning experiences. ^20^

The strong correlation between cognitive skill growth and platform usage (r = 0.62, p < 0.01) supports studies that show AI personalization has a positive effect on academic achievement. In support of this, Wang et al. showed that adaptive learning systems powered by AI have the ability to improve student performance dramatically. ^15^

Qualitative comments by our participants, for instance, "I enjoyed how it adapted to my pace," demonstrate tenets of self-determination theory, in which autonomy increases intrinsic motivation. This is as opposed to research indicating that AI technologies, through providing individualized and interactive learning opportunities, can profoundly improve student motivation and motivation.

Nonetheless, rural students experienced some difficulties due to time limitations ("It was difficult to find time with our normal classes") and software problems ("Sometimes the app crashed"). These are representative of larger infrastructural differences between urban and rural school environments. It is found that restricted availability of technology and stable internet connections in rural areas can play a hindrance to the effective use of AI-based learning tools. ^21^

The disparities in cognitive and language gains observed between rural (13.2 and 7.8 points, respectively) and urban students (17.5 and 10.5 points) also underscore the role of infrastructural issues in determining the outcomes of learning. These disparities need to be bridged to ensure equal benefits of AI-based educational interventions across different demographic segments.

Faculty attitudes in our research indicate a balanced perspective on AI implementation in dental education. Educators recognized AI as an effective complement to conventional pedagogy, observing, "It's a great tool to supplement teaching," but insisted that it should not substitute for clinical hands-on practice. This view is consistent with overall trends in dental education, in which technological innovations are welcomed to improve learning, but the inimitable worth of clinical training continues to take precedence. In accordance with this, a report by Dashti et al. highlights that although AI-based applications in healthcare have shown efficacy, clinical training remains central to ensuring competent dental professionals. The need for extra training to make proper use of AI in curricula was an emergent need expressed by several faculty members with statements like, "We need more training to integrate it into our lectures." This is consonant with reports from a multicenter study stating that faculty members tend to possess low AI literacy and have different perceptions regarding the place of AI in education. ^22^

AI has been viewed as a revolutionary tool for teaching and learning, while others express concerns about its potential misuse and impact on critical thinking skills. ^23^ Our mixed-methods study uncovered a synergy between quantitative gains and qualitative advantages, like enhanced accessibility. Technical difficulties were also reported, which indicate the requirement of strong support mechanisms to enable easy integration. These findings align with an increasing body of evidence highlighting the necessity of comprehensive training and support for instructors to implement AI tools effectively in dental education. ^24^^,^^25^

Our subgroup analysis indicates the critical influence of context on the success of educational interventions. Students from urban areas showed greater improvement, which could be the result of better internet connectivity and technological familiarity. In contrast, students from rural areas were troubled, likely by sparse connectivity and technology. These findings align with past literature regarding India's digital divide in education. A study on the present situation of the digital divide in India's education system identified that just 31% of the rural population makes use of the internet, whereas 67% of the urban population uses the internet, which indicates enormous imbalances in digital access. ^26^ The resultant absence of technological access automatically affects education, disadvantaging students from poor families. ^27^^,^^28^ In spite of these challenges, evidence suggests that AI-based personalized learning systems can improve learning outcomes in rural and urban areas. An experiment in Karnataka, India, showed substantial gains in mathematics proficiency among students using AI systems, with rural experimental group scores jumping from a pre-test mean of 62.5 to a post-test mean of 78.3, and urban experimental group scores improving from 68.2 to 83.9. ^29^ These results imply that although AI interventions are helpful across varied settings, the size of their impact can be subject to infrastructural and situational factors. 

In line with the step-by-step guide described by Skamagki et al. ^29^ this study demonstrates Mixed-Methods Integration at the design, methods, and interpretation levels. The quantitative findings (e.g., statistically significant gains in post-test and CAT scores) are supported and contextualized by the qualitative themes that describe students’ increased confidence and faculty perspectives on practical implementation. This explicit linking of strands enhances the explanatory power and relevance of the study’s conclusions.

This study’s faculty feedback aligns with broader trends reported internationally, which show that educators recognize the potential of AI to enhance student-centered learning but also call for structured faculty development initiatives. ^30^^,^^31^ Future implementation should incorporate regular faculty training and institutional support to maximize AI’s benefits while addressing practical barriers in real-world educational contexts.

The findings of the study suggest that the AI-based personalized learning system meaningfully enhanced both cognitive skills and domain-specific language confidence in Oral Medicine and Radiology. This is consistent with studies in other medical education contexts showing that adaptive AI systems improve knowledge retention and language proficiency by providing personalized feedback and contextual assistance. ^3^^,^^32^ By integrating such features into a Hybrid PBL format, the present study demonstrates the practical potential of AI to support learners in multilingual, resource-diverse educational settings.

The qualitative data was useful as a supplement to the quantitative data in showing that the use of the AI modules resulted in improved student independence and critical thinking skills. The feedback provided by the educators revealed both the strengths and weaknesses of the process and stressed the importance of educator training and proper resources. While the study considered knowledge retention, critical thinking, language skills, and student engagement simultaneously to give an overview of the entire process, it is recommended that each aspect be studied independently in future studies.

These findings are significant concerning the use of competency-based dental education and early clinical exposure. Improvement in cognitive skills will facilitate the process of developing clinical reasoning and will be helpful in making correct diagnoses and proper treatment plans.

Language proficiency improvements will be beneficial for patient-dentist interactions, as a crucial element in achieving quality care. The multilingual capabilities will be particularly useful in multicultural contexts such as India, helping students understand complex notions and interact successfully with patients. Positive correlation between engagement levels and academic achievements indicates that the use of AI tools contributes to fostering active and effective learning. In institutions with limited clinical experience, AI-assisted training may play an additional part in the training process. From an educational viewpoint, performance assessment and feedback are the key elements of competency-based teaching, which may be ensured by using AI technology. At the same time, inconsistencies across different institutional contexts suggest that infrastructural gaps should be filled out.

Overall, the implementation of AI-powered personalized learning strategies can significantly contribute to enhancing the process of learning, as well as improving clinical competencies and patient communication abilities. However, sufficient teacher training is required.

There were several limitations that had to be taken into account. Even though the system used during the research allowed for multilingualism, there could have been some differences in terms of regional culture and communication style that were not adequately covered. This problem can be solved by involving the assistance of faculty members and integrating culturally adaptive content and region-specific case libraries in further projects.

Three institutions were chosen for participation in the experiment, but they were geographically distributed. However, students' residence backgrounds were not stratified, which may also influence their learning experience and technological opportunities. Furthermore, no assessment of cost-effectiveness was conducted, which will be critical in rural settings.

While the system offered its users a multilingual approach, certain differences in communication might have impacted students' engagement and understanding. Despite the significant improvement in students' ability to use English as the technical language in the field, the results have to be understood through the prism of multilingualism typical of Indian dental education. Finally, even though AI provides considerable help in the process, it cannot serve as a replacement for faculty supervision and peer collaboration in developing professional communication skills.

## CONCLUSION

This study provides preliminary evidence that integrating AI-based personalized learning with Hybrid PBL can improve knowledge retention and technical language confidence in a diverse, multilingual dental student cohort. However, the results should be interpreted within the context of the unique linguistic diversity of the participating colleges and should not be generalized beyond similar settings without further research.

## References

[1] Annamma LM, Varma SR, Abuttayem H, Prasad P, Azim SA, Odeh R, George BT, Nair C, Karobari MI (2024). Current challenges in dental education- a scoping review. BMC Med Educ.

[2] Dental Council of India (2021). Annual Report 2020-21.

[3] Wang W, Bi X, Zhu Y, Li X (2020). Reforming teaching methods by integrating dental theory with clinical practice for dental students. PeerJ.

[4] Rao A (2021). Linguistic diversity, education, and democracy in India prospects and challenges. Bull Chelyabinsk State Univ.

[5] Alqahtani T, Badreldin HA, Alrashed M, Alshaya AI, Alghamdi SS, Bin Saleh K, Alowais SA, Alshaya OA, Rahman I, Al Yami MS, Albekairy AM (2023). The emergent role of artificial intelligence, natural learning processing, and large language models in higher education and research. Res Social Adm Pharm.

[6] Krishnan G, Singh S, Pathania M, Gosavi S, Abhishek S, Parchani A, Dhar M (2023). Artificial intelligence in clinical medicine catalyzing a sustainable global healthcare paradigm. Front Artif Intell.

[7] Kabra L, Santhosh VN, Sequeira RN, Ankola AV, Coutinho D (2023). Dental curriculum reform in India: Undergraduate students' awareness and perception on the newly proposed choice based credit system. J Oral Biol Craniofac Res.

[8] (2021). Oral Health in America: Advances and Challenges.

[9] Kamalov F, Santandreu Calonge D, Gurrib I (2023). New Era of Artificial Intelligence in Education Towards a Sustainable Multifaceted Revolution. Sustainability.

[10] Mohan P, Kumar R (2019). Strengthening primary care in rural India Lessons from Indian and global evidence and experience. J Family Med Prim Care.

[11] Guo J, Li B (2018). The Application of Medical Artificial Intelligence Technology in Rural Areas of Developing Countries. Health Equity.

[12] Elendu C, Amaechi DC, Okatta AU, Amaechi EC, Elendu TC, Ezeh CP, Elendu ID (2024). The impact of simulation-based training in medical education A review. Medicine (Baltimore).

[13] Kolb DA (2015). Experiential learning: experience as the source of learning and development. Second edition.

[14] Salian A, Sanal Kumar S, Natarajan S, Lewis AJ, Manaktala N, Naik DG, Boaz K, Kp N, Yellapurkar S (2022). Performance of Dental Students in Understanding and Retention of Oral Pathology Concepts A Comparative Analysis of Traditional versus Live-Field Teaching Methods. ScientificWorldJournal.

[15] Wang X, Huang R, Sommer M, Pei B, Shidfar P, Rehman MS (2024). The Efficacy of Artificial Intelligence-Enabled Adaptive Learning Systems From 2010 to 2022 on Learner Outcomes A Meta-Analysis. J Educ Comput Res.

[16] Junaidi J (2020). Artificial intelligence in EFL context rising students' speaking performance with Lyra virtual assistance. Int J Adv Sci Technol Rehabil.

[17] Wei L (2023). Artificial intelligence in language instruction impact on English learning achievement, L2 motivation, and self-regulated learning. Front Psychol.

[18] Fang Q, Reynaldi R, Araminta AS, Kamal I, Saini P, Afshari FS, Tan SC, Yuan JC, Qomariyah NN, Sukotjo C (2025). Artificial Intelligence (AI)-driven dental education Exploring the role of chatbots in a clinical learning environment. J Prosthet Dent.

[19] Jegede OO (2024). Artificial intelligence and English language learning exploring the roles of AI-driven tools in personalizing learning and providing instant feedback. Universal Library of Languages and Literatures.

[20] Zaharuddin Zaharuddin, Yu C, Yao G (2024). Enhancing student engagement with AI-driven personalized learning systems. Int Trans Educ Technol.

[21] Maita KC, Maniaci MJ, Haider CR, Avila FR, Torres-Guzman RA, Borna S, Lunde JJ, Coffey JD, Demaerschalk BM, Forte AJ (2024). The Impact of Digital Health Solutions on Bridging the Health Care Gap in Rural Areas A Scoping Review. Perm J.

[22] Dashti M, Ghasemi S, Khurshid Z (2024). Integrating Artificial Intelligence in Dental Education An Urgent Call for Dedicated Postgraduate Programs. Int Dent J.

[23] Al-Zubaidi SM, Muhammad Shaikh G, Malik A, Ul Zain, Abideen M, Tareen J, Alzahrani NSA, Ahmed Siddiqui A (2024). Exploring Faculty Preparedness for Artificial Intelligence-Driven Dental Education A Multicentre Study. Cureus.

[24] Thurzo A, Strunga M, Urban R, Surovková J, Afrashtehfar KI (2023). Impact of Artificial Intelligence on Dental Education A Review and Guide for Curriculum Update. Education Sciences.

[25] Claman D, Sezgin E (2024). Artificial Intelligence in Dental Education Opportunities and Challenges of Large Language Models and Multimodal Foundation Models. JMIR Med Educ.

[26] Tayade M, Chavan V (2023). Digital Divide in Indian Education System: An Analysis of Present Scenario. Education and Society.

[27] Golden AR, Srisarajivakul EN, Hasselle AJ, Pfund RA, Knox J (2023). What was a gap is now a chasm Remote schooling, the digital divide, and educational inequities resulting from the COVID-19 pandemic. Curr Opin Psychol.

[28] Korkmaz Ö, Erer E, Erer D (2022). Internet access and its role on educational inequality during the COVID-19 pandemic. Telecomm Policy.

[29] Roy K, Swargiary Khritish (2024). The Impact of AI-Driven Personalized Learning on Mathematics Achievement and Student Engagement in Rural vs. Urban Schools in Karnataka, India.

[30] Skamagki G, King A, Carpenter C, Wåhlin C (2024). The concept of integration in mixed methods research a step-by-step guide using an example study in physiotherapy. Physiother Theory Pract.

[31] Schwendicke F, Samek W, Krois J (2020). Artificial Intelligence in Dentistry Chances and Challenges. J Dent Res.

[32] Schlenz MA, Michel K, Wegner K, Schmidt A, Rehmann P, Wostmann B (2020). Undergraduate dental students&apos; perspective on the implementation of digital dentistry in the preclinical curriculum a questionnaire survey. BMC Oral Health.

[33] Li J, Zong H, Wu E, Wu R, Peng Z, Zhao J, Yang L, Xie H, Shen B (2024). Exploring the potential of artificial intelligence to enhance the writing of english academic papers by non-native english-speaking medical students - the educational application of ChatGPT. BMC Med Educ.

